# Clinical evaluation of two new unshielded and shielded silicon diode detectors in external photon beam radiation therapy: SunSILICON and SunSILICON P

**DOI:** 10.1002/acm2.70576

**Published:** 2026-04-19

**Authors:** Teresa J. Anders, Veronika Flatten, Mohamad Alissa, David Towle, Andy Murray, Ann‐Britt Schönfeld, Erik Alquist, Olivier Evrard, Jeff Hildreth, Ndimofor Chofor, Gerhard Wessing, Charbel Habib, Damian Czarnecki, Andreas A. Schönfeld

**Affiliations:** ^1^ Radiologie Vechta Vechta Germany; ^2^ Universität Bremen Bremen Germany; ^3^ Sun Nuclear Corp. Melbourne Florida USA; ^4^ CDT‐West ‐ Centrum für Diagnostik und Therapie Cologne Germany; ^5^ Mirion Technologies Olen Antwerp Belgium; ^6^ MyMichigan Health Midland Michigan USA; ^7^ Institut für Medizinische Physik und Strahlenschutz Technische Hochschule Mittelhessen Gießen, Hesse Germany

**Keywords:** dosimetry, energy dependence, shielded diode, silicon diode, small field dosimetry, unshielded diode

## Abstract

**Background:**

Advanced external photon beam radiotherapy requires dosimetry detectors with high spatial resolution and minimal perturbation effects. Silicon diode detectors are widely used due to their high sensitivity and small sensitive volumes, but fluence perturbation effects and energy dependence can affect performance and depend on detector construction. Shielded and unshielded configurations are used to address these challenges, but their clinical performance and limitations require systematic evaluation.

**Purpose:**

This study aims to evaluate the clinical performance of two newly developed silicon diode detectors—SunSILICON (unshielded) and SunSILICON P (shielded)—for relative dosimetry in external photon beam radiotherapy. The detectors’ response behaviors are compared to established detectors across the full clinical range of field sizes and beam energies, focusing on percentage depth dose (PDD) and lateral beam profile measurements.

**Methods:**

PDD and lateral beam profile measurements across the full range of clinical field sizes were performed on Varian TrueBeam and Elekta Versa HD clinical linear accelerators with photon energies ranging from 4 to 25 MV, as well as a Nordion Eldorado 6 ^60^Co unit, using various types of motorized water phantoms. Comparative measurements were performed with ionization chambers, as well as other silicon and diamond detectors. Over 600 scans were analyzed, and gamma analysis was applied to assess agreement using 0.5%/0.5 mm for PDDs and 1%/1 mm for lateral beam profiles. Angular dependence, detector sensitivity and the effective point of measurement (EPOM) were determined in solid phantoms.

**Results:**

SunSILICON and SunSILICON P demonstrated excellent agreement with established detectors within their specified field size ranges. Gamma passing rates exceeded 99% for most comparisons, with minor deviations in large fields or at field size limits. The shielded SunSILICON P showed reduced energy dependence in large fields compared to the unshielded version, while SunSILICON showed superior performance in small fields. Both detectors exhibited minimal angular dependence (<1% for clinically relevant angles) and negligible intra‐type variation. The measured EPOMs matched calculated values within uncertainty. The combination of both detectors enables comprehensive coverage of clinical dosimetry needs.

**Conclusions:**

SunSILICON and SunSILICON P provide reliable, high‐resolution dosimetry for external photon beam radiotherapy across a broad range of clinical scenarios. Their performance is comparable to or exceeds that of established silicon and diamond detectors, with the shielded version particularly suited for larger fields due to its reduced energy dependence. Together, these detectors offer a robust solution for clinical relative dosimetry.

## 1 INTRODUCTION

Advanced external beam radiotherapy with photon radiation, using field sizes from small beamlets to large extended fields, typically 0.5 cm × 0.5 cm to 40 cm × 40 cm, requires universally applicable dosimetry detectors with high spatial resolution. An established option for relative dosimetry is the silicon diode detector, since silicon and water feature a relatively linear stopping power ratio over the energy range of interest.^1^ Owing to its high density and comparatively low ionization energy required for the generation of charge carriers, silicon exhibits a radiation‐induced current density that is approximately 18 000 times greater than that of air contained in ionization chambers.^2^, ^3^ For that reason, significantly smaller sensitive volumes can be realized with silicon‐based detectors enabling a reduction of the volume averaging effect, which can be relevant in the penumbra region of lateral beam profile scans or when measuring small field output factors.^4^, ^5^, ^6^ Silicon, however, shows an increased response to keV photon energies.^3^, ^4^ The significance of this property increases at larger field sizes and greater measurement depths, where the occurrence of low‐energy scattered radiation increases. Its effect can be reduced by incorporating high‐Z material around the silicon diode acting as a filter for low energy photons. Such a detector configuration is also referred to as shielded diode or photon diode, often labeled in the product name with a “P” or “X” to mark the photon use.

However, high‐density, high‐Z components like the shielding component or silicon itself enhance the detector's fluence perturbation effects and contribute to the magnitude of the small field output correction factors. The underlying medium and density effects, and therein the role of the material's mean excitation energy is actively discussed in literature.^3^, ^6^, ^7^, ^8^, ^9^, ^10^, ^11^, ^12^, ^13^, ^14^ Consequentially, the use of unshielded silicon diodes is advised in small photon fields and electron beams.

Two new silicon diode detectors, the unshielded 1048 SunSILICON and the shielded 1049 SunSILICON P (both Sun Nuclear Corp., Melbourne, USA) were developed for relative dosimetry in external beam radiotherapy. Both detectors feature a newly developed silicon diode model^15^ and their housings are made from water‐equivalent HE Solid Water (Sun Nuclear Corp., USA) aiming to minimize fluence perturbations.

The objective of this study is to evaluate the performance of these silicon diode detectors in relative dosimetry applications of clinical photon beams by comparison of percentage depth dose (PDDs) measurements and lateral beam profile measurements to other established detectors. In addition, the nominal response to ^60^Co radiation, the angular dependence of the response, and the effective point of measurement (EPOM) of the two detectors were determined experimentally. Further technical aspects concerning the diode, such as reproducibility, linearity with dose, the speed of response, impedance and leakage characteristics, accumulated dose dependency, temperature dependency, pulse repetition frequency dependence, and instantaneous dose rate dependency are discussed in detail in a separate study.^15^

## 2 MATERIALS AND METHODS

### 2.1 SunSILICON and SunSILICON P

The detectors both contain the same p‐type silicon diode with an active volume of 0.053 mm^3^. The detector housing is made of HE Solid Water, near‐water equivalent epoxy, and otherwise only differs by the shielding component in SunSILICON P. The detector's entrance window is perpendicular to its long axis with the center positioned 4.05 mm from the housing tip. The nominal water‐equivalent build‐up depth is 1.25 mm. The active volume of the diode is a disc with a radius of 0.75 mm and a depth of 0.03 mm. Both detectors are operated in photovoltaic mode, meaning no bias voltage is applied. As typical for an unshielded diode, SunSILICON is intended to be used in small photon and clinical electron beams. SunSILICON P, as a shielded diode, is intended for general use in photon beams.

**FIGURE 1:**
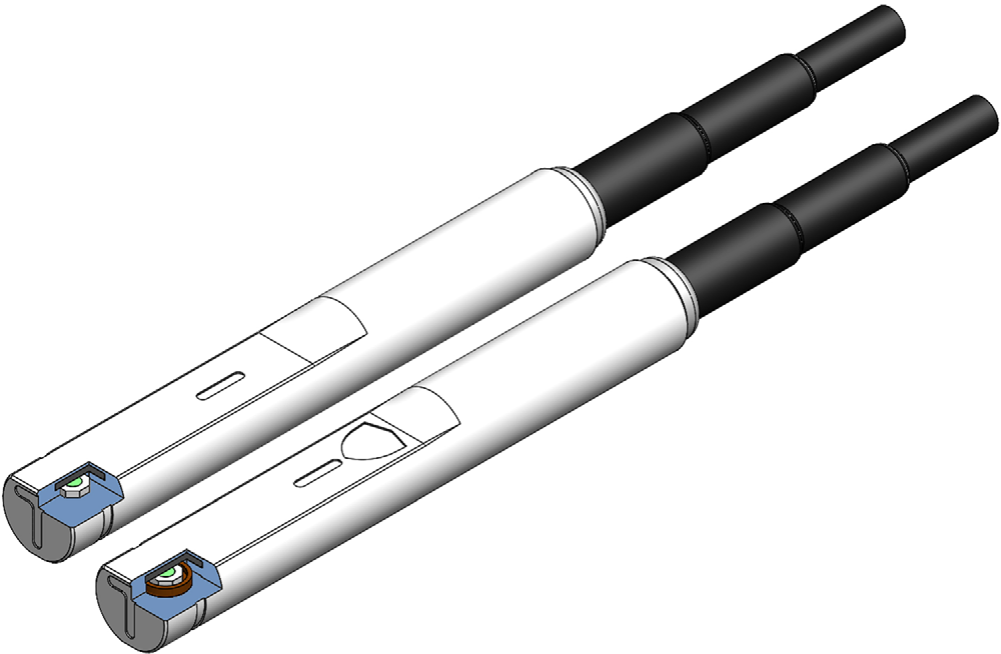
Drawings of the unshielded silicon diode detector SunSILICON (left) and the shielded silicon diode detector SunSILICON P (right). Not to scale. Not all details are shown.

The field size range for photon beams, as indicated by the manufacturer, is 0.4–10 cm for SunSILICON and 2–40 cm for SunSILICON P. Field sizes are expressed in terms of the width of the square field. The respective detector construction drawings are shown in Figure 1 (top) for the SunSILICON and in Figure 1 (bottom) for the SunSILICON P.

### 2.2 Measurement setup

An overview of measurements, measurement conditions, and detectors is listed in Table 1.

**TABLE 1:** Overview of tests performed with the associated setup, radiation quality, machine output, field size, detectors, and test specifics.

Test	Setup	Radiation qualities	Dose monitor	Field sizes (cm^2^)	Detectors	Test specifics
T1.1 Percentage depth dose curves	Water phantom	4 MV 6 MV FFF 6 MV 10 MV FFF 15 MV 25 MV	N/A	0.5 × 0.5 to 40 × 40	1 × EDGE Detector 1 × PTW microSilicon 1 × PTW microSilicon X 1 × PTW microDiamond 1 × SunSILICON 1 × SunSILICON P	PDDs obtained on the central beam axis at a source to surface distance (SSD) of 90 cm. Scans taken from 30 cm depth upwards to water surface.
T1.2 Cross‐plane profiles	Water phantom	4 MV 6 MV FFF 6 MV 10 MV FFF 15 MV 25 MV	N/A	0.5 × 0.5 to 40 × 40	1 × SNC125c 1 × EDGE Detector 1 × PTW microSilicon 1 × PTW microSilicon X 1 × PTW microDiamond 1 × SunSILICON 1 × SunSILICON P	Cross‐plane profiles measured in 10 cm depth with SSD of 90 cm.
T1.3 Effective point of measurement (EPOM)	HE Solid Water slab phantom	6 MV	100 MU	20 × 20	2 × SunSILICON 2 × SunSILICON P	Test method according to of Looe et al.^16^, ^19^: Comparison of stepwise TPR measurements against a known reference (SNC350p,^17^). Experimental data used to verify calculated EPOM value.
T1.4 Sensitivity	1D Scanner	^60^Co	3 min	10 × 10	1 × SNC600c 3 × SunSILICON 3 × SUNSILICON P	Reference dosimetry according to TRS‐398.^18^ EPOM of silicon diode detectors was matched to that of SNC600c.
T1.5 Angular dependence of the response a) Yaw (Rotation) b) Roll & Pitch (Tilt)	a) SunSCAN 3D b) Spherical phantom	6 MV	a) 200 MU b) 100 MU	3 × 3	3 × SunSILICON 3 × SunSILICON P	a) Rotation about the symmetry‐axis of the diode chip (yaw axis of the detector stem). b) Rotation about the pitch and roll axes of the detector stem. All axes aligned to the center of the sensitive volume.

#### 2.2.1 Irradiation devices

A Varian TrueBeam (Varian Medical Systems, Palo Alto, USA) clinical linear accelerator (linac) with nominal photon beam energies of 6, 10, 10 FFF (Flattening‐Filter Free), and 15 MV, an Eldorado 6 (Nordion, Ottawa, Canada) ^60^Co teletherapy unit, and an Elekta Versa HD (Elekta AB, Stockholm, Sweden) with nominal photon energies of 4 and 25 MV served as irradiation devices.

#### 2.2.2 Phantoms

PDDs and lateral beam profiles were measured with motorized water phantoms, including SunSCAN 3D (Sun Nuclear Corp., USA), BluePhantom 2 (IBA Dosimetry, Schwarzenbruck, Germany), and BeamScan (PTW, Freiburg, Germany), depending on the availability at the respective measurement sites; see Table 1, T1.1 and T1.2. Detectors were set up using holders and setup routines recommended by the manufacturers. All measurements were taken at a source‐to‐surface‐distance (SSD) of 90 cm, with the lateral beam profiles obtained at a measurement depth of 10 cm. Sufficient time was allowed to let the water temperature equilibrate to ambient conditions.

The effective point of measurement was determined following the methodology presented by Looe et al.^16^, ^17^ using an HE Solid Water slab phantom (Sun Nuclear Corp., USA); see Table 1, T1.3. A 1D Scanner (Sun Nuclear Corp., USA) for detector positioning and ^60^Co radiation was used to determine the detector sensitivity (response); see Table 1, T1.4. The angular dependence of the response was measured in a spherical, rotationally symmetric PMMA phantom with customized inserts for SunSILICON and SunSILICON P; see Table 1, T1.5.

#### 2.2.3 Test conditions

In alignment with standard measurement procedures (if applicable), sufficient build‐up and backscatter material was used, background was corrected for, and air density correction was applied to point measurements conducted with ionization chambers. The TRS 398^18^ dosimetry protocol was applied for absolute dose measurements. Specific considerations are listed in Table 1.

#### 2.2.4 Detectors

Most measurements were performed with one of three new SunSILICON or SunSILICON P detectors. When intra‐type variability was of interest, all three detectors of each type were investigated. Other detectors used for comparison included the 60019 microDiamond, 60023 microSilicon, 60022 microSilicon X (all PTW), 1041 SNC125c, and 1118 EDGE Detector (both Sun Nuclear Corp.).

#### 2.2.5 Field sizes for PDDs and lateral beam profiles

The field size ranges in which PDD and lateral beam profile measurements were intercompared were guided by the specifications of the compared detectors. For example, SunSILICON and SunSILICON P measurements were compared to SNC125c measurements in field sizes of 4 cm to 10 and 4 to 40 cm, respectively. While the comparison to a reference class ionization chamber is considered the gold standard, the microDiamond was incorporated as a reference in small fields, where volume averaging becomes increasingly significant. Thus, comparisons to microDiamond were made in field sizes up to 10 cm. Since microSilicon and SunSILICON, as well as microSilicon X and SunSILICON P are detectors of the same type (unshielded and shielded silicon diodes), the detectors were compared over the whole field size range, regardless of field size specifications. In total, over 600 scans were intercompared. Most of the intercomparisons were made at nominal beam energies of 4, 6, 15, and 25 MV. All other beam energies (6 MV FFF, 10 MV, 10 MV FFF) included at least two lateral beam profiles and at least one PDD per detector.

## 3 RESULTS

### 3.1 Percentage depth dose curves

To initially assess the general performance of SunSILICON and SunSILICON P, PDD curves were compared to those obtained with established unshielded and shielded silicon diode detectors, PTW microSilicon and microSilicon X.^12^, ^20^, ^21^, ^22^, ^23^, ^24^, ^25^, ^26^, ^27^ A strict gamma criterion of 0.5% dose difference, a distance‐to‐agreement of 0.5 mm and no low dose threshold was applied after aligning the PDDs in the build‐up region to account for detector setup uncertainties.

The comparisons of SunSILICON and microSilicon were made in field sizes of (0.5, 0.6, 0.8, 1, 1.2, 2, 3, 4, 6, 8, 10, 15, 20, and 40 cm) at beam energies of 6 and 15 MV. Gamma passing rates exceeded 99% for all measured field sizes and energies with only two exceptions showing 98.8% and 98.5% in field sizes of 1 and 40 cm at 6 MV. SunSILICON P and microSilicon X were compared in field sizes of (1, 1.5, 2, 3, 4, 6, 7, 8, 10, 15, 20, and 40 cm) for both energies, respectively. Gamma passing rates exceeded 99% for all measured field sizes and both energies.

Since all silicon diodes are subject to energy dependence of the response, PDD comparisons to an SNC125c ionization chamber were made over the energy range of 4–25 MV. For SunSILICON, 20 different beam settings in the field size range of 4–10 cm were intercompared. For SunSILICON P, the different beam settings in the field size range of 4–40 cm resulted in 24 intercomparisons. Representative examples from the analysis are shown for a 6 MV photon beam in

In addition, PDD comparisons to a PTW microDiamond were made in the field size range of 2–10 cm over the energy range of 4–25 MV resulting in 20 different beam settings for SunSILICON and 14 different beam settings for SunSILICON P. Figure 3 shows representative examples of PDD comparisons against a PTW microDiamond detector for beam energies ranging from 4 to 25 MV at 10 cm field size.

**FIGURE 2:**
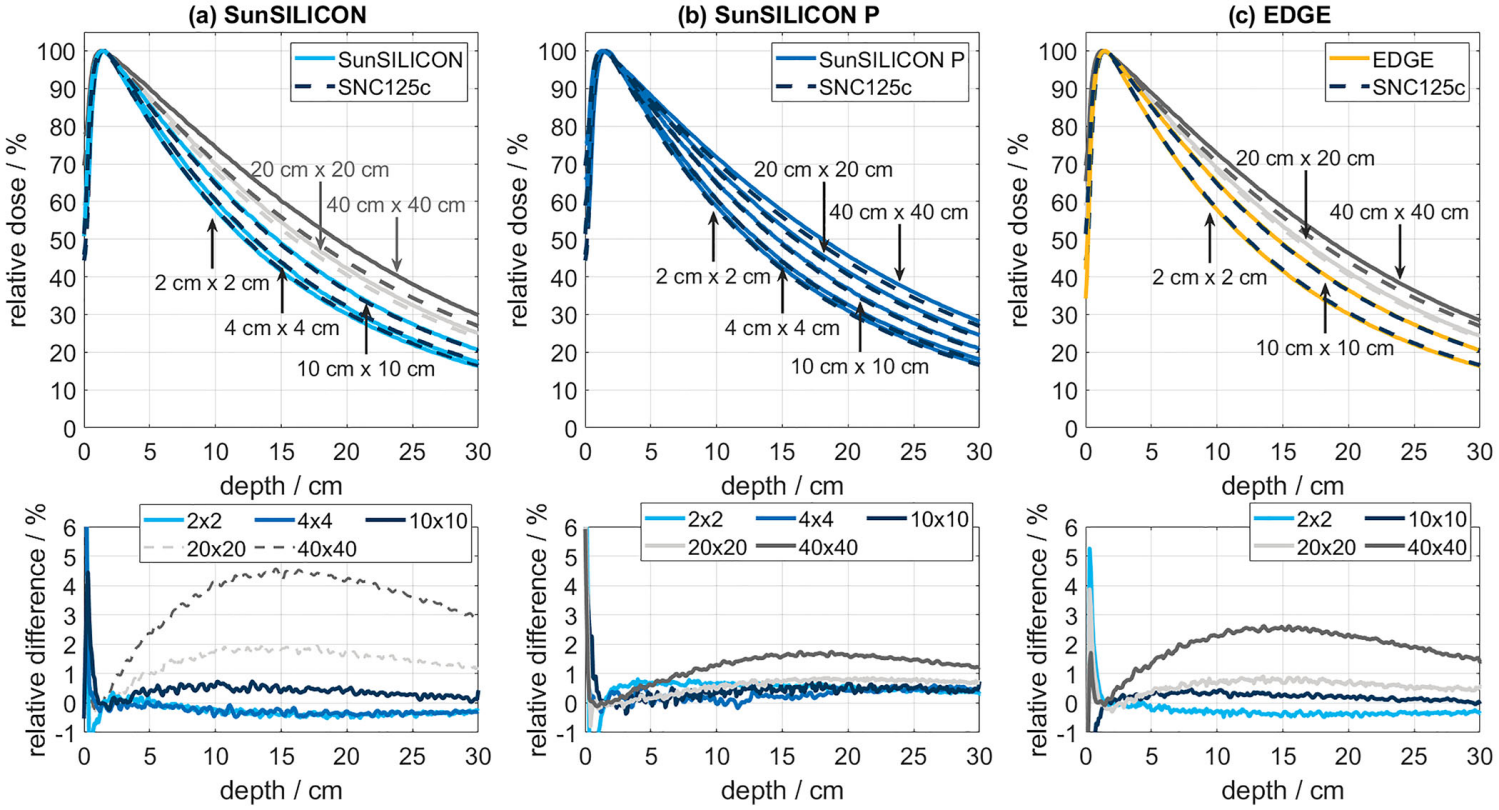
PDD curves (6 MV) measured with SNC125c compared to SunSILICON (a), SunSILICON P (b), and EDGE Detector (c) with the corresponding difference plots (bottom row). Differences in the corresponding beam quality specifiers obtained from the PDDs are shown in Table 2.

Since PDD measurements are performed to determine the beam quality specifier TPR_20,10_, according to TRS 398,^18^ or D_10_, according to AAPM TG‐51^28^ in clinical practice, Table 2 lists the deviations of the beam quality specifiers obtained with SunSILICON and SunSILICON P from those obtained with the SNC125c corresponding to the PDDs shown in Figure 2.

**TABLE 2:** Differences in TPR_20,10_ and D_10_ for PDDs measured with SunSILICON (left) and SunSILICON P (right) with respect to those measured with SNC125c. Shaded cells are field sizes outside of the specifications of SunSILICON.

SunSILICON vs. SNC125c			SunSILICON P vs. SNC125c		
Field size (cm^2^)	ΔTPR_20,10_	ΔD_10_	Field size (cm^2^)	ΔTPR_20,10_	ΔD_10_
2 × 2	0.004	0.3%	2 × 2	0.002	0.5%
4 × 4	0.001	0.4%	4 × 4	0.007	0.0%
10 × 10	0.002	0.6%	10 × 10	0.007	0.3%
20 × 20	0.010	1.8%	20 × 20	0.008	0.4%
40 × 40	0.023	4.0%	40 × 40	0.011	1.3%

### 3.2 Cross‐plane profiles

Cross‐plane beam profiles measured with SunSILICON and SunSILICON P were compared to those obtained with microSilicon and microSilicon X using a gamma criterion of 1% dose difference, 1 mm distance‐to‐agreement and 10% low dose threshold. The criterion was chosen in consideration of the steeper dose gradients and the threshold to apply a uniform cut‐off method. The comparisons were made in field sizes of (0.5, 0.6, 0.8, 1, 1.2, 2, 3, 4, 6, 8, 10, 15, 20, and 40 cm) at beam energies of 6 and 15 MV.

In the comparison of SunSILICON to microSilicon, gamma passing rates were 100% for all measured field sizes with only four exceptions showing 97.3%, 98.3%, 98.7%, and 94.7% in field sizes of 6 cm (6 MV), 15, 20, and 40 cm (all 15 MV). Notably, neither detector is specified for field sizes larger than 10 cm.

The comparison of SunSILICON P with microSilicon X in field sizes of (1, 1.5, 2, 3, 4, 6, 8, 10, 15, 20, 40 cm) and at beam energies of 6 and 15 MV yielded gamma passing rates of 100% in all cases.

In addition, lateral beam profile comparisons to a PTW microDiamond were made over the energy range of 4–25 MV in the field size range of 0.5–10 cm resulting in 20 different beam settings for SunSILICON and in the field size range of 1–10 cm resulting in 14 different beam settings for SunSILICON P. Passing rates were 100% for SunSILICON, with only three exceptions showing 98.1%, 97.5%, and 97.2% for field sizes of 4 cm (15 MV), 10 cm (25 MV), and 2 cm (25 MV). Passing rates were 100% for SunSILICON P, without exception.

SunSILICON and SunSILICON P were also compared to SNC125c and EDGE Detector measurements. Notably, the different magnitudes of volume averaging introduced by silicon diodes and a compact ionization chamber such as SNC125c prevents a meaningful gamma comparison in the penumbra region. Figures 4 and 5 show representative half profiles of a 6 MV photon beam measured at various field sizes. Figure 5 sets focus on large field scans conducted with SunSILICON P.

### 3.3 Effective point of measurement

The water equivalent depths of HE Solid Water (0.23 mm), epoxy (0.74 mm), paint (0.17 mm), and some proprietary materials (total of 0.11 mm) add up to a total water equivalent EPOM depth of 1.25 mm as measured from the surface of the detector housing. The measured EPOM of SunSILICON and SunSILICON P was found to be 1.2 ± 0.3 and 1.45 ± 0.3 mm, respectively, following the methodology presented by Looe et al.^16^, ^19^ Measurements match the calculated EPOM within measurement uncertainty. The main contributors to the measurement uncertainty are setup reproducibility, EPOM location of the reference detector,^17^ and thickness of the HE Solid Water slabs.

### 3.4 Sensitivity

The response to ^60^Co radiation was measured to be 36.98 and 36.99 nC/Gy for two unirradiated 1048 SunSILICON detectors and 38.52, 38.53, and 38.37 nC/Gy for three unirradiated 1049 SunSILICON P detectors. A third 1048 SunSILICON unit under test had previously been exposed to 3–4 kGy of electron radiation and showed a response of 36.64 nC/Gy.

### 3.5 Angular dependence of the response

SunSILICON and SunSILICON P showed an angular response dependence of less than 0.1% for all yaw angles (rotation about the symmetry‐axis of the diode). The angular response with respect to tilt angles (pitch and roll) was determined by a general cosine fit model, where the response deviates by less than 1% for angles below ±28° (SunSILICON) and ±21° (SunSILICON P), respectively (Figure 7).

## 4 DISCUSSION

### 4.1 Percentage depth dose measurements

Regarding the energy dependence of the response of detectors, it is important to note that spectral softening or hardening with depth on the central beam axis is field‐size dependent (Figure 8a). While spectral softening occurs in field sizes larger than 10 cm, spectral hardening is observed in field sizes smaller than 10 cm. Notably, the mean photon energy is relatively constant with respect to depth at 10 cm field size.^29^ Spectral softening or hardening is due to varying contributions of low‐energy scatter components in small and large field sizes.^29^, ^30^, ^31^ At small field sizes, spectral hardening is caused by the Compton effect, since enhanced lateral scattering at lower photon energies steers low‐energy scatter away from the central beam axis. With increasing field sizes and increasing depth, however, an increasing occurrence of low‐energy Compton‐scattered photons decreases the mean photon energy.^31^

In consequence, the energy dependence of the detector response can significantly affect the shape of the measured PDD curve, and thus, the numeric value of the displayed beam quality specifiers. The underlying reason is the interplay of the fluence spectrum variation and the interaction coefficients of the detector components and its active medium (Figure 8d). Fluence perturbation effects of detector components have been thoroughly discussed in literature in various contexts.^6^, ^14^, ^22^, ^27^, ^33^, ^34^, ^35^ Other influencing quantities adding uncertainty to PDD measurements are dose rate dependence of the response, due to varying dose rate with depth and field size,^15^ and beam axis alignment, specifically in small radiation fields. Therefore, it is important to understand the range of beam parameters in which a detector is well behaved.

As visible in the left panel of Figure 2, PDD measurements conducted with unshielded silicon diodes such as SunSILICON agree well with detectors known for their energy independence, such as compact ionization chambers, at field sizes of 10 cm or less because the mean photon energy increases with depth (Figure 8a) and the photon attenuation is linear with respect to water for increasing photon energies (Figure 8d). At field sizes larger than 10 cm, however, the decreasing mean photon energy causes an increasing over‐response of the detector with depth, which is due to the increased attenuation of silicon to low photon energies (Figure 8d). The near‐perfect agreement of SunSILICON and microSilicon data underlines this commonality between unshielded silicon diode detectors.

Accordingly, Table 2 confirms good correspondence of beam quality specifiers measured with SunSILICON in field sizes of 10 cm or less. At larger field sizes, the beam quality specifier TPR_20,10_ is more robust with respect to energy dependent effects than $D_{10}$, due to a relatively consistent signal shift at measurement depths of 10 and 20 cm (bottom of Figure 2a), respectively. A contributing factor to this phenomenon may be the reduced back‐scatter at the bottom of the water phantom.^36^, ^37^ Since the PDD is normalized to $d_{\max}$, $D_{10}$ is directly affected by the observed signal shift at a measurement depths of 10 cm.

This energy dependence of the detector response is reduced by the shielding component of SunSILICON P, as shown in Figure 2b. The difference between the SNC125c measurement and SunSILICON P is less than 1% for field sizes smaller than 20 cm, and less than 2% for a field size up to 40 cm. As AAPM's task group report TG 142^38^, ^39^ recommends the constancy check of the beam quality specifier $D_{10}$ with a tolerance limit of 1% against the baseline, the baseline measurement should be performed with the same detector type. The comparison to the EDGE Detector (Figure 2c) reveals the importance of the shielding design: While EDGE Detector is also considered a shielded diode, the effect of the shielding is much smaller. SunSILICON P and microSilicon X produced near identical measurement results.

While the inherent build‐up of SunSILICON and SunSILICON P of 1.25 mm is slightly larger than that of the EDGE Detector,^40^ the accessible build‐up region of the PDD curve is captured correctly, as can be seen in Figure 2. Calculated and measurement‐based determinations of the EPOM agreed within measurement uncertainty. Notably, the dose difference plots in the bottom row of Figure 2 spike in the build‐up region, due to the steep dose gradient.

**FIGURE 3:**
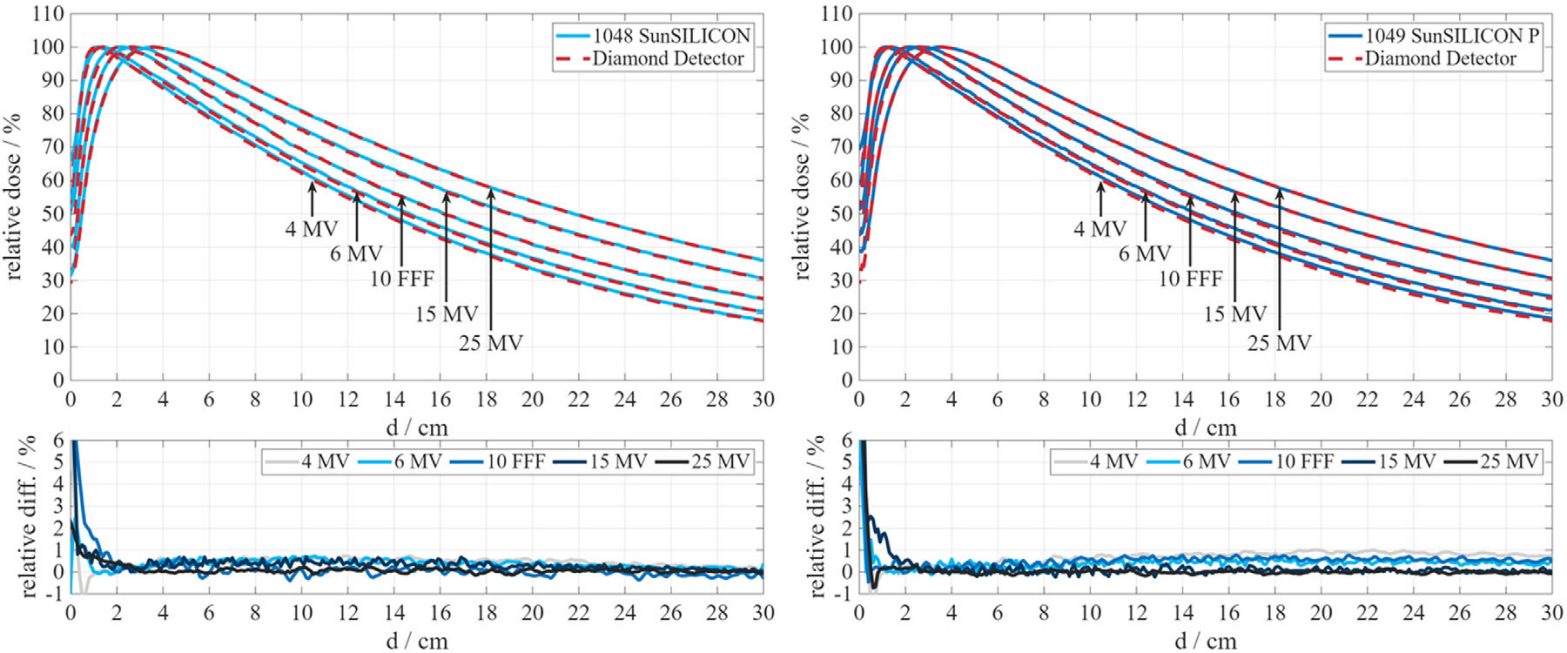
PDDs measured with PTW microDiamond compared to SunSILICON (left) and SunSILICON P (right) with the corresponding difference plots (bottom row).

Figure 3 shows PDDs measured with SunSILICON and SunSILICON P at various beam energies ranging from 4 to 25 MV in comparison to the PTW microDiamond. While the mean photon energy is consistent with depth at a field size of 10 cm, the dose rate in terms of dose per pulse varies proportionally to the PDD value. The consistent agreement of the PDDs (bottom row of Figure 3) confirms the small dose rate dependence of the response reported for SunSILICON and SunSILICON P.^15^

### 4.2 Lateral beam profile measurements

The mean photon energy is relatively consistent across a lateral beam profile, but drops significantly outside of the penumbra (Figure 8b). The exceptions are large photon fields, where a lateral gradient of the mean photon energy can occur, due to the reduction of lateral backscatter, as well as the geometry of the flattening filter, in case of flattened beams (Figure 8c). Larger photon fields also show a higher dose in the tails of the lateral beam profile, due to the enhanced flux of scattered photons.

**FIGURE 4:**
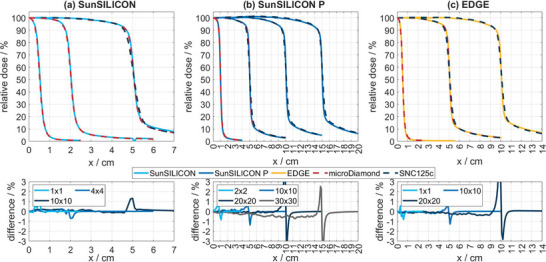
Half cross‐plane profiles for 6 MV photon fields of varying field size measured with (a) microDiamond, SunSILICON and SNC125c; (b) microDiamond, SunSILICON P, and SNC125c; and (c) EDGE Detector, microDiamond and SNC125c. The 20 cm field size in (c) exceeds the field size specification of the EDGE Detector (gray line). The bottom row shows the respective difference plots to microDiamond for field sizes smaller than 10 cm and to SNC125c for larger field sizes. Note the different scales on the x‐axis.

**FIGURE 5:**
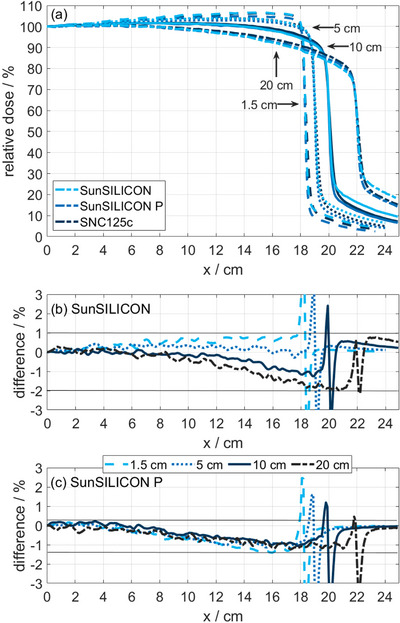
(a) Half cross‐plane profiles of a 6 VM, 40 cm × 40 cm photon field measured at 1.5, 5, 10, and 20 cm depth in water using SNC125c, SunSILICON, and SunSILICON P. (b) Difference plot of the SunSILICON and SNC125c measurements. (c) Difference plot of the SunSILICON P and SNC125c measurements. The legend between (b) and (c) applies to both panels. Note that the displayed field size is outside of the specified field size range of SunSILICON.

In consequence, the effect of energy dependence on lateral beam profiles measured with an unshielded silicon diode, such as SunSILICON, is negligible in small photon fields, as apparent from the comparison to microDiamond in Figure 4a. Likewise, no significant effect of volume averaging could be observed (bottom panel of Figure 4a). At a field size of 10 cm, the tails of the lateral beam profiles exhibit a slight over‐response, which becomes more pronounced with increasing field size and is evident from the comparison to profile scans with SNC125c. Notably, the measurement results produced by SunSILICON and microSilicon were near identical. The over‐response in the tails is mitigated by the shielding component in SunSILICON P (Figures 4b and 5), while the EDGE Detector's shielding component is less effective at larger field sizes (Figure 4c). SunSILICON P and microDiamond profiles match within measurement uncertainty, except for the tails, where SunSILICON P shows a slightly lower signal.

The potential significance of the energy dependence of unshielded silicon diode detectors in lateral beam profile measurements of very large photon fields is demonstrated in Figure 5. With respect to an ion chamber measurement, the response diverges significantly in the outer part of the field plateau (Figure 5b), exhibiting an apparent over‐response in shallow depths and an apparent under‐response in deeper depths. This behavior is, in fact, an artifact of the diode response behavior at the respective normalization points on the central beam axis. The causation of this behavior is revealed in Figure 8c showing that the lateral distribution of mean photon energy with respect to the normalization point is depth dependent, where the mean photon energy increases towards the field edges at deeper depths and decreases towards the field edges at shallow depths. The normalization process to the field center, the lateral distribution of the mean photon energy, and the interplay of photon fluence and attenuation of the detector components (Figure 8d) lead to the observed detector behavior.

**FIGURE 6:**
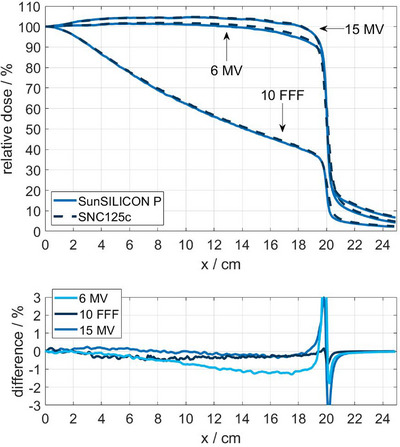
Top panel: Half cross‐plane profiles of a 40 cm × 40 cm photon field measured at 10 cm depth in water with nominal beam energies of 6 MV, 10 MV FFF, and 15 MV using SNC125c and SunSILICON P. Bottom panel: Difference plot of the SunSILICON P and SNC125c measurements.

MicroSilicon X exhibited the same behavior observed for SunSILICON P and produced near‐identical results. Notably, this effect is only pronounced in large photon fields, where a significant lateral gradient of the mean photon energy occurs. This may be influenced by the nominal beam energy and the geometry of the flattening filter (Figure 6), in addition to the scanning depth.

The effect is mitigated by the shielding component of SunSILICON P (Figure 5c). While there is a residual offset between SunSILICON P and the SNC125c, it is consistent across all investigated profile depths. As shown in Figure 6, the shielded diode SunSILICON P shows consistent agreement with the ion chamber measurement in both large flattened and large unflattened fields.

**FIGURE 7:**
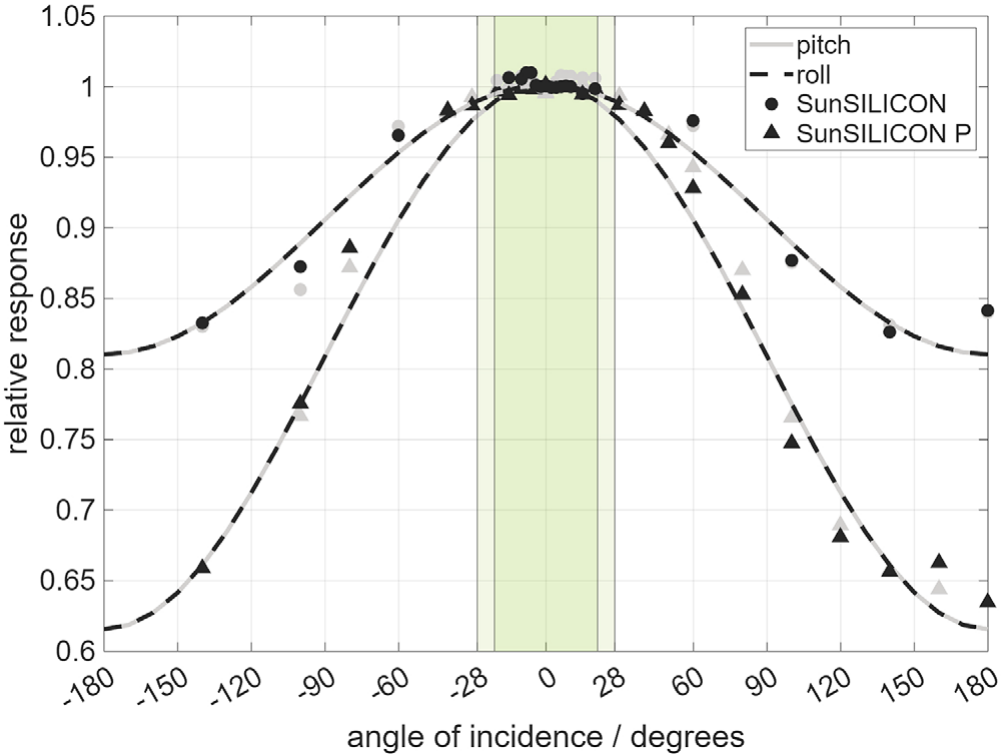
SunSILICON and SunSILICON P's angular dependence of the response as a function of the beam incidence angle. Data was fitted with a general cosine model. Values are within 1% from the reference value measured at a beam angle of 0° for beam angles of ±28° (SunSILICON) and ±21° (SunSILICON P) as highlighted by the shaded area.

Another contributing factor to the measurement uncertainty of a lateral beam profile is the detector's angular dependence of the response, since the angle of incidence from the point source at the Bremsstrahlung's target changes with the lateral offset of the detector. For example, at a source‐to‐detector distance (SDD) of 100 cm, lateral detector offsets of 5 or 20 cm from the central beam axis correspond to angles of incidence of 2.9° and 11.3°. SunSILICON and SunSILICON P demonstrate an angular dependence of less than 1% for angles of incidence up to ±28° and ±21°, respectively, see Figure 7. At a SDD of 100 cm, this translates to total accessible scanning widths of 106 and 77 cm.

**FIGURE 8:**
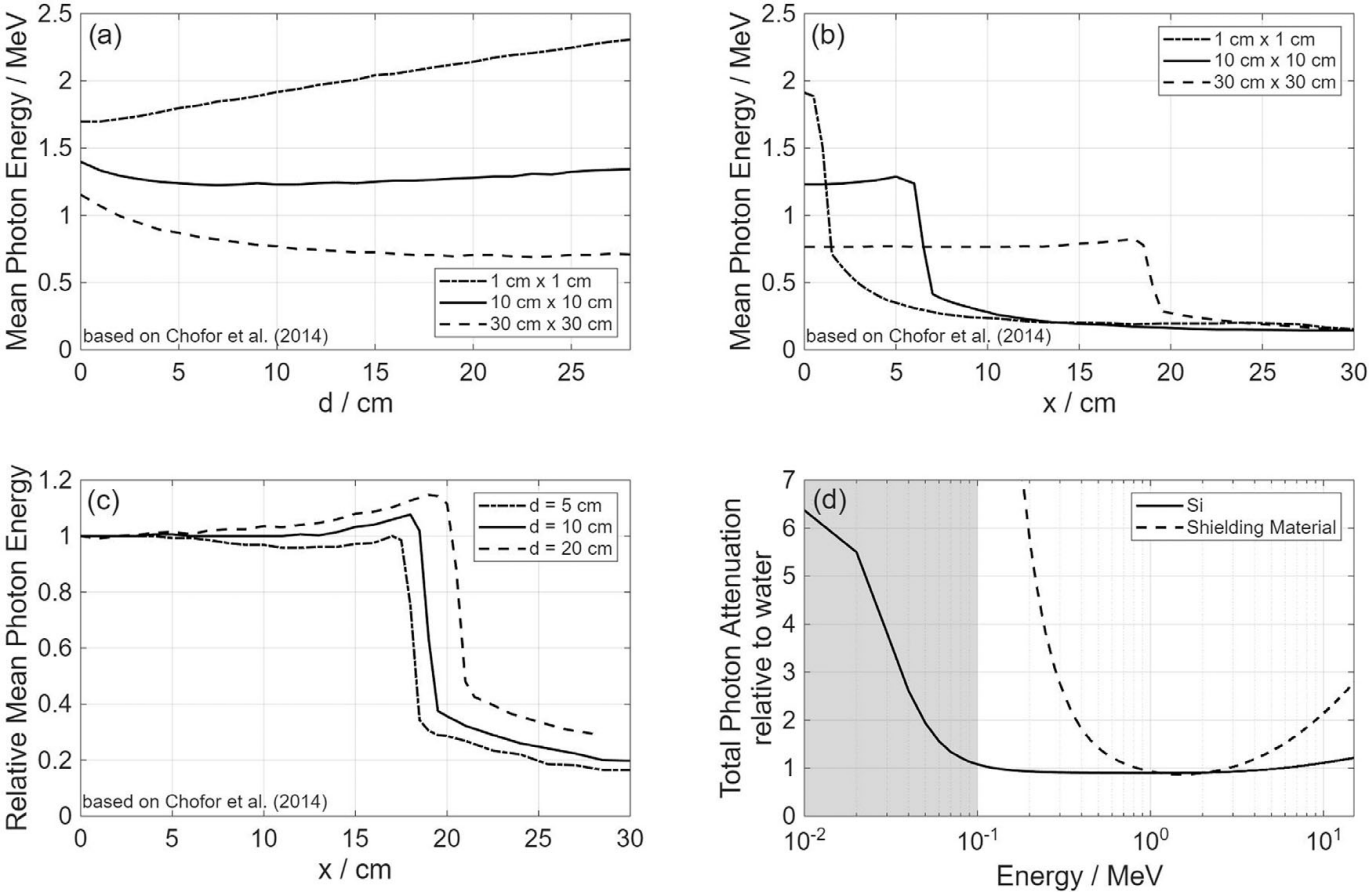
(a) Mean photon energy distribution on the central beam axis of a 6 MV beam with respect to depth in water d and field size. (b) Mean photon energy distribution of a 6 MV beam at 10 cm depth in water with respect to the off‐axis position x and field size. (c) Normalized mean photon energy distribution of a 30 cm × 30 cm, 6 MV photon field at 5, 10, and 20 cm depth in water with respect to the off‐axis position x. Data shown in (a), (b), and (c) is based on Chofor et al. 2014.^29^ (d) Total photon attenuation^32^ of Si and the main shielding component normalized to the total photon attenuation of water. The shaded region highlights photon energies, to which a silicon‐based detector overresponds. The dashed line indicates the attenuation effect of a shielding component.

### 4.3 General remarks

The evaluation of the response of the detectors showed a maximum intra‐type‐variation of less than 0.5%, suggesting a consistent build across different detectors. Lateral beam profile and PDD measurements were performed for a wide range of beam settings, as indicated in Table 1. While only a representative subset of data could be visualized, no contradictory findings were made.

While Figures 2a and 5b show the deficiencies of unshielded silicon diodes in large photon fields, it should be emphasized that these detectors are typically specified only for field sizes of 10 cm or less. Since small photon fields are subject to spectral hardening, neither the effect of silicon's inherent overresponse to low photon energies, nor the fluence perturbation introduced by the shielding component affect the measurement performance, which shows in the similarity of the lateral beam profiles and PDDs acquired with SunSILICON and microDiamond (Figures 2a and 4a). Furthermore, the construction of an unshielded silicon diode, as well as the favorable stopping power ratio of silicon and water allow the usage of unshielded silicon diodes in electron beam dosimetry.

## 5 CONCLUSION

The performance of SunSILICON and SunSILICON P in clinical relative dosimetry was studied and the physical effects underlying typical silicon diode detector behavior were discussed. The detectors exhibit excellent performance in their respective field size range as verified by comparison to microDiamond, microSilicon, microSilicon X, and SNC125c across a broad range of field sizes and beam energies. As a pair, SunSILICON and SunSILICON P can cover the full range of relative dosimetry measurements conducted in radiation therapy.

## AUTHOR CONTRIBUTIONS

Teresa J. Anders conducted the measurements on the Elekta linac and drafted the manuscript. Mohamad Alissa contributed to the shielding design. David Towle, Andy Murray, Ann‐Britt Schönfeld, Erik Alquist, and Charbel Habib conducted the measurements on the Varian linac. Olivier Evrard and Jeff Hildreth designed and tested the diode. Veronika Flatten contributed to the design of the study and analysis. Ndimofor Chofor contributed data for the energy distribution analysis. Gerhard Wessing collected the measurements on the Elekta linac together with Teresa J. Anders. Damian Czarnecki contributed to the study and detector design. Andreas A. Schönfeld supervised the study design and analysis and substantially revised the manuscript. All authors revised the manuscript and approved the final version.

## CONFLICT OF INTEREST STATEMENT

D.T., A.M., A.‐B.S., E.A., O.E., J.H., V.F., N.C., and A.S. are employees of Mirion. The remaining authors declare that the research was conducted in the absence of any commercial or financial relationships that could be construed as a potential conflict of interest.

## ETHICS STATEMENT

This article does not involve any studies on human participants/animals conducted by the authors.

## Data Availability

Authors will share data upon request.

## References

[1] Seltzer SM , Berger MJ . Evaluation of the collision stopping power of elements and compounds for electrons and positrons. Int J Appl Radiat Isot. 1982;33:1189–1218. doi:10.1016/0020-708X(82)90244-7

[2] Yorke E , Alecu R , Ding L , et al. TG 62: Diode In Vivo Dosimetry for Patients Receiving External Beam Radiation Therapy . Medical Physics Publishing; 2005.

[3] Andreo P , Burns DT , Nahum AE , Seuntjens J , Attix FH . Fundamentals of Ionizing Radiation Dosimetry. John Wiley & Sons; 2017.

[4] IAEA . TRS 483: Dosimetry of Small Static Fields Used in External Beam Radiotherapy. 2017.

[5] Crop F , Reynaert N , Pittomvils G , et al. The influence of small field sizes, penumbra, spot size and measurement depth on perturbation factors for microionization chambers. Phys Med Biol. 2009;54:2951. doi:10.1088/0031-9155/54/9/024 19384005

[6] Bouchard H , Seuntjens J , Duane S , Kamio Y , Palmans H . Detector dose response in megavoltage small photon beams. I. Theoretical concepts. Med Phys. 2015;42:6033–6047. doi:10.1118/1.4930053 26429279

[7] Andreo P , Benmakhlouf H . Role of the density, density effect and mean excitation energy in solid‐state detectors for small photon fields. Phys Med Biol. 2017;62:1518‐1532. doi:10.1088/1361-6560/aa562e 28036305

[8] Looe HK , Harder D , Poppe B . Understanding the lateral dose response functions of high‐resolution photon detectors by reverse Monte Carlo and deconvolution analysis. Phys Med Biol. 2015;60:6585‐6607. doi:10.1088/0031-9155/60/16/6585 26267311

[9] Andreo P . The physics of small megavoltage photon beam dosimetry. Radiother Oncol. 2018;126:205‐213. doi:10.1016/j.radonc.2017.11.001 29191457

[10] Fenwick JD , Kumar S , Scott AJ , Nahum AE . Using cavity theory to describe the dependence on detector density of dosimeter response in non‐equilibrium small fields. Phys Med Biol. 2013;58:2901‐2923. doi:10.1088/0031-9155/58/9/2901 23574749

[11] Looe HK , Delfs B , Poppinga D , Jiang P , Harder D , Poppe B . The ‘cutting away’ of potential secondary electron tracks explains the effects of beam size and detector wall density in small‐field photon dosimetry. Phys Med Biol. 2017;63:015001. doi:10.1088/1361-6560/aa9b46 29148434

[12] Schönfeld AB , Poppinga D , Kranzer R , et al. Technical note: characterization of the new microSilicon diode detector. Med Phys. 2019;46:4257‐4262. doi:10.1002/mp.13710 31309594 PMC6852691

[13] Griessbach I , Lapp M , Bohsung J , Gademann G , Harder D . Dosimetric characteristics of a new unshielded silicon diode and its application in clinical photon and electron beams. Med Phys. 2005;32:3750‐3754. doi:10.1118/1.2124547 16475774

[14] Schönfeld AA , Alissa M , Towle D , et al. Field output correction factors and perturbation factor analysis for novel SunSILICON and SunSILICON P silicon diode detectors. J Appl Clin Med Phys. 2026;27:e14712. 10.1002/acm2.70632 PMC13239861 42219627

[15] Anders T , Flatten V , Evrard O , et al. Characterization of two new unshielded and shielded silicon diode detectors for external beam radiation therapy: SunSILICON and SunSILICON P. Zeitschrift für Medizinische Physik. 2025. doi:10.1016/j.zemedi.2025.12.005 41444080

[16] Looe HK , Harder D , Poppe B . Experimental determination of the effective point of measurement for various detectors used in photon and electron beam dosimetry. Phys Med Biol. 2011;56:4267‐4290. doi:10.1088/0031-9155/56/14/005 21701053

[17] Schönfeld AB , Schönfeld AA , Looe HK , Poppe B , de Wilde RL . Experimental determination of the recombination correction factor kS for SNC 125c, SNC 350p and SNC 600c ionization chambers in pulsed photon beams. Z Med Phys. 2020;30(4):300‐304. doi:10.1016/j.zemedi.2020.03.001 32278506

[18] IAEA . TRS 398 Rev1: Absorbed Dose Determination in External Beam Radiotherapy. 2024.

[19] Looe HK , Chofor N , Djouguela A , Harder D , Poppe B . Accurate Experimental Determination of the Effective Point of Measurement of Radiation Detectors, Dreiländertagung ‘Medizinphysik 2007’ (Bern) (2007).

[20] Akino Y , Das IJ , Fujiwara M , et al. Characteristics of microSilicon diode detector for electron beam dosimetry. J Radiat Res. 2021;62:1130–1138. doi:10.1093/jrr/rrab085 34559877

[21] Akino Y , Okamura K , Das IJ , et al. Technical note: characteristics of a microSilicon X shielded diode detector for photon beam dosimetry. Med Phys. 2021;48:2004‐2009. doi:10.1002/mp.14639 33278843

[22] Delbaere A , Younes T , Simon L , Khamphan C , Vieillevigne L . Field output correction factors and electron fluence perturbation of the microSilicon and microSilicon X detectors. Phys Med Biol. 2022;67:08NT01. doi:10.1088/1361-6560/ac5e5e 35294937

[23] Francescon P , Kilby W , Noll JM , Satariano N , Orlandi C . Small field dosimetry correction factors for circular and MLC shaped fields with the CyberKnife M6 System: evaluation of the PTW 60023 microSilicon detector. Phys Med Biol. 2020;65:01NT01. doi:10.1088/1361-6560/ab6154 31829983

[24] Georgiou G , Kumar S , Würfel J , et al. The PTW microSilicon diode: performance in small 6 and 15 MV photon fields and utility of density compensation. Med Phys. 2021;48:8062‐8074. doi:10.1002/mp.15329 34725831

[25] McGrath AN , Golmakani S , Williams TJ . Determination of correction factors in small MLC‐defined fields for the Razor and microSilicon diode detectors and evaluation of the suitability of the IAEA TRS‐483 protocol for multiple detectors. J Appl Clin Med Phys. 2022;23:e13657. doi:10.1002/acm2.13657 35652320 PMC9278669

[26] Poppinga D , Kranzer R , Ulrichs AB , et al. Three‐dimensional characterization of the active volumes of PTW microDiamond, microSilicon, and Diode E dosimetry detectors using a proton microbeam. Med Phys. 2019;46:4241‐4245. doi:10.1002/mp.13705 31292964 PMC6851623

[27] Weber C , Kranzer R , Weidner J , et al. Small field output correction factors of the microSilicon detector and a deeper understanding of their origin by quantifying perturbation factors. Med Phys. 2020;47:3165‐3173. doi:10.1002/mp.14149 32196683 PMC7496769

[28] Almond PR , Biggs PJ , Coursey BM , et al. TG 51: protocol for clinical reference dosimetry of high‐energy photon and electron beams. Med Phys. 1999;26:1847‐1870. doi:10.1118/1.598691 10505874

[29] Chofor N , Harder D , Poppe B . Supplementary values of the dosimetric parameters kNR and Em for various types of detectors in 6 and 15 MV photon fields. Z Med Phys. 2014;24:27‐37. doi:10.1016/j.zemedi.2013.04.001 23642543

[30] Chofor N , Harder D , Poppe B . Non‐reference condition correction factor kNR of typical radiation detectors applied for the dosimetry of high‐energy photon fields in radiotherapy. Z Med Phys. 2012;22:181‐196. doi:10.1016/j.zemedi.2012.05.001 22658451

[31] Chofor N , Harder D , Willborn K , Ruhmann A , Poppe B . Low‐energy photons in high‐energy photon fields–Monte Carlo generated spectra and a new descriptive parameter. Z Med Phys. 2011;21:183‐197. doi:10.1016/j.zemedi.2011.02.002 21530198

[32] Hubbell JH , Seltzer SM . Tables of X‐Ray mass attenuation coefficients and mass energy‐absorption coefficients 1 keV to 20 MeV for elements Z = 1 to 92 and 48 additional substances of dosimetric interest. national inst. of standards and technology‐pl; 1995. https://www.osti.gov/biblio/76335

[33] Bouchard H , Kamio Y , Palmans H , Seuntjens J , Duane S . Detector dose response in megavoltage small photon beams. II. Pencil beam perturbation effects. Med Phys. 2015;42:6048‐6061. doi:10.1118/1.4930798 26429280

[34] Hartmann GH , Andreo P , Kapsch RP , Zink K . Cema‐based formalism for the determination of absorbed dose for high‐energy photon beams. Med Phys. 2021;48:7461‐7475. doi:10.1002/mp.15266 34613620

[35] Roers J , Czarnecki D , Alissa M , Zink K . Spectral analysis of Monte Carlo calculated fluence correction and cema conversion factors for high‐energy photon beams at different depths. Front Phys. 2023;10:1075514. doi:10.3389/fphy.2022.1075514

[36] Schoenfeld AA , Harder D , Poppe B , Chofor N . Water equivalent phantom materials for (1)(9)(2)Ir brachytherapy. Phys Med Biol. 2015;60:9403‐9420. doi:10.1088/0031-9155/60/24/9403 26579946

[37] Carlsson Tedgren A , Carlsson GA . Influence of phantom material and dimensions on experimental 192Ir dosimetry. Med Phys. 2009;36:2228‐2235. 19610312 10.1118/1.3121508

[38] Klein EE , Hanley J , Bayouth J , et al. TG 142: quality assurance of medical accelerators. Med Phys. 2009;36:4197‐4212. doi:10.1118/1.3190392 19810494

[39] Hanley J , Dresser S , Simon W , et al. TG 198: an implementation guide for TG 142 quality assurance of medical accelerators. Med Phys. 2021;48:e830–e885, doi:10.1002/mp.14992 34036590

[40] Sun Nuclear Corporation . Edge Detector User Guide . Sun Nuclear Corp., 2025.

